# Correlation of serum DKK1 level with skeletal phenotype in children with osteogenesis imperfecta

**DOI:** 10.1007/s40618-024-02380-9

**Published:** 2024-05-14

**Authors:** Y. Wang, J. Hu, L. Sun, B. Zhou, X. Lin, Q. Zhang, O. Wang, Y. Jiang, W. Xia, X. Xing, M. Li

**Affiliations:** Department of Endocrinology, Key Laboratory of Endocrinology, National Health and Family Planning Commission, Peking Union Medical College Hospital, Chinese Academy of Medical Sciences and Peking Union Medical College, Shuaifuyuan No. 1, Beijing, 100730 Dongcheng District China

**Keywords:** Osteogenesis imperfect, Dickkopf-1, Bone mineral density, Bone turnover markers

## Abstract

**Purpose:**

We aim to detect serum DKK1 level of pediatric patients with OI and to analyze its relationship with the genotype and phenotype of OI patients.

**Methods:**

A cohort of pediatric OI patients and age-matched healthy children were enrolled. Serum levels of DKK1 and bone turnover biomarkers were measured by enzyme-linked immunosorbent assay. Bone mineral density (BMD) was measured by Dual-energy X-ray absorptiometry. Pathogenic mutations of OI were detected by next-generation sequencing and confirmed by Sanger sequencing.

**Results:**

A total of 62 OI children with mean age of 9.50 (4.86, 12.00) years and 29 healthy children were included in this study. The serum DKK1 concentration in OI children was significantly higher than that in healthy children [5.20 (4.54, 6.32) and 4.08 (3.59, 4.92) ng/mL, *P* < 0.001]. The serum DKK1 concentration in OI children was negatively correlated with height (r = − 0.282), height Z score (r = − 0.292), ALP concentration (r = − 0.304), lumbar BMD (r = − 0.276), BMD Z score of the lumbar spine and femoral neck (r = − 0.32; r = − 0.27) (all *P* < 0.05). No significant difference in serum DKK1 concentration was found between OI patients with and without vertebral compression fractures. In patients with spinal deformity (22/62), serum DKK1 concentration was positively correlated with SDI (r = 0.480, *P* < 0.05). No significant correlation was observed between serum DKK1 concentration and the annual incidence of peripheral fractures, genotype and types of collagen changes in OI children.

**Conclusion:**

The serum DKK1 level was not only significantly elevated in OI children, but also closely correlated to their skeletal phenotype, suggesting that DKK1 may become a new biomarker and a potential therapeutic target of OI.

**Supplementary Information:**

The online version contains supplementary material available at 10.1007/s40618-024-02380-9.

## Introduction

Osteogenesis imperfecta (OI) is a rare monogenic hereditary osteopathy with an incidence of 1 in 15,000 to 20,000 live births, characterized by bone fragility and multiple bone fractures [1]. OI is caused by alteration in synthesis and post-translational modification of type I collagen due to multiple genetic mutations [2, 3]. Recent studies have shown that the severity of OI phenotype is not only related to abnormal type I collagen metabolism, but also closely linked to changes in osteoblast activity [4, 5], of which the mechanism is not yet clear.

As is well known, WNT/β-catenin pathway plays an important role in regulation of differentiation and activity of osteoblasts. *WNT1* mutation can lead to severe OI through leading to inactivation of WNT pathway and osteoblasts, suggesting that WNT pathway may be involved in the pathological processes of OI [6, 7]. Previous studies indicated that the natural inhibitors of the WNT pathway, including sclerostin, frizzled protein, and Dickkopf-1 (DKK1), produced marked effects on the differentiation, maturation and activity of osteoblasts [8]. DKK1 can block the binding of WNT to the specific cell surface receptors low-density lipoprotein receptor-related protein 5/6 (LRP5/6), and reduce the level of β-catenin in osteoblasts, then reducing the differentiation and maturation of osteoblasts [9]. However, it is unclear whether there is a change in serum DKK1 level in OI patients and whether it is related to OI phenotype.

Therefore, we detect the serum DKK1 level in pediatric patients with OI and analyze its relationship with BMD, fracture incidence, bone turnover makers, and the pathogenic genotype of OI, and to determine whether DKK1 has value as a novel biomarker and therapeutic target of OI.

## Methods

### Subjects

Patients less than 18 years old with OI were recruited from the Department of Endocrinology, Peking Union Medical College Hospital (PUMCH), from April 2017 to October 2023. The inclusion criteria of OI patients were as follows: (1) a history of at least one fracture under minor trauma during childhood and an age- and gender-adjusted BMD Z-score less than -2.0 at lumbar spine (LS) or proximal femur before any anti-osteoporosis therapy; (2) presence of blue sclera or dentinogenesis imperfecta and a family history of OI [10, 11]. The exclusion criteria were as follows: with other genetic or metabolic bone diseases, with other disease that could affect bone metabolism, ongoing treatment with glucocorticoids, anti-epileptic drugs, bisphosphonates, denosumab, teriparatide, etc., and with liver or kidney dysfunction.

Age-matched healthy children who underwent physical examinations at PUMCH were included as control. This study was approved by the Scientific Ethics Committee of PUMCH (JS-3545D), and informed consents were obtained from legal guardian of each OI patient and healthy children.

### Phenotype assessment

The following phenotypic information of OI patients was collected: age of OI onset, age of confirmed OI, frequency and sites of bone fracture, skeletal malformations and extra-skeletal manifestations, including blue sclera, dentinogenesis imperfecta, hearing loss, joint ligament laxity, and muscle atrophy. Height and weight were measured using a Harpenden measuring instrument (Seritex, Inc., East Rutherford, NJ, USA). For patient who was unable to stand, body length in the supine position was measured. Height and weight Z scores for OI patients at different ages and gender were calculated according to the normal reference values for Chinese children [12].

Clinical fractures included nonvertebral fractures and symptomatic vertebral fractures, which were reported by the patients or their legal guardians and confirmed by X-ray films. A semiquantitative assessment of vertebral compression fracture (VCFs) was performed by radiologists at PUMCH using Genant classification [13]. The semiquantitative Spinal Deformity Index (SDI) provides a comprehensive evaluation of spinal fracture status, considering both the number and severity of fractures. Each vertebra is visually graded on a scale of 0 to 3, representing no fracture, mild, moderate, or severe fracture, and SDI is calculated by summing these grades across all vertebrae from T4 to L4 [14]. Scoliosis was confirmed by posterior-anterior radiographs and defined as a Cobb angle higher than 10° [15]. The annual incidence of peripheral fractures was calculated by dividing the total number of peripheral fractures by the duration of disease. Areal BMD at the lumbar spine (LS) 1–4, the femoral neck (FN) and the total hip was measured using dual-energy X-ray absorptiometry (DXA, GE Lunar Prodigy Advance, USA) and analyzed by software compatible with pediatric data. Calibration and quality checks were completed daily using the DXA equipment. Patients with vertebral compression fractures or significantly deformation were excluded from the lumbar BMD analysis. The BMD Z scores of the LS and FN of children and adolescents were calculated according to the normal BMD reference values for Asian children [16, 17].

The disease phenotype exhibits significant heterogeneity, including the mildest form (type I), the most severe form among surviving patients (type III), an intermediate form between type I and type III (type IV), and the unique type with interosseous membrane calcification of the forearm and/or hypertrophic callus (type V) [18, 19]. The perinatal lethality (type II) OI was not included in this study.

### Determination of the serum DKK1 and biochemical marker concentration

Fasting blood samples of OI patients and healthy controls were obtained at 8:00–10:00 in the morning. The serum DKK1 concentration was measured by enzyme-linked immunosorbent assay (ELISA) (Cat. No. DKK100B, R&D systems, USA), which was completed by Key Laboratory of Endocrinology, National Health and Family Planning Commission, PUMCH. The minimum detection value was 0.948 pg/mL, the intra-assay coefficients of variation (CV) was 1.8–2.9%, and the inter-assay CV was 7.7–8.7%.

The serum concentrations of osteoprotegerin (OPG) and sclerostin were measured by enzyme-linked immunosorbent assay (ELISA) (Cat. No. SEA108Hu, Cloud-Clone Corp, China and Cat. No. BI-20472, BIOMEDICA, Austria). The minimum detection value of OPG and sclerostin were 0.059 ng/mL and 1.3 pmol/L, respectively. The intra-assay CV were ≤ 10%and ≤ 1% for OPG and sclerostin measurement, respectively. The inter-assay CVs were ≤ 12% and ≤ 5% for OPG and sclerostin detection, respectively.

The serum levels of calcium (Ca), phosphorus (P) and alkaline phosphatase (ALP, a bone formation marker) were measured using an automatic analyser (ADVIA 1800, Siemens, Germany). The serum levels of β-isomerized carboxy-telopeptide of type I collagen (β-CTX, a bone resorption marker), procollagen I N-terminal peptide (P1NP, a bone formation marker), 25-hydroxyvitamin D (25OHD), and intact parathyroid hormone (PTH) were assessed using an automated electrochemiluminescence system (E170, Roche Diagnostics, Switzerland). All the biochemical indicators were detected by clinical central laboratory of PUMCH.

### Detection of pathogenic mutations in OI patients

Genomic DNA was extracted from peripheral leukocytes of OI patients using a DNA extraction kit (QIAamp DNA, Qiagen, Frankfurt, Germany), which was sequenced using targeted next-generation sequencing (NGS) (Illumina HiSeq2000 platform, Illumina, Inc., San Diego, CA, USA) [20]. The targeted NGS panel included all known candidate genes of OI, including *COL1A1, COL1A2, IFITM5, SERPINF1, CRTAP, P3H1, PPIB, SERPINH1, FKBP10, PLOD2, BMP1, SP7, TMEM38B, WNT1, CREB3L1, SPARC, MBTPS2, P4HB, SEC24D* and *PLS3*, and 708 other skeletal disease-associated candidate genes [21]. The pathogenicity of the detected variants was classified according to the 2015 guidelines of the American College of Medical Genetics and Genomics/Association for Molecular Pathology (ACMG/AMP) [22]. The pathogenic mutations identified by NGS were validated by polymerase chain reaction (PCR) and Sanger sequence (3730 DNA Analyser, Applied Biosystems, Foster City, CA, USA).

According to genetic patterns, OI patients were divided into autosomal dominant inheritance (AD) and non-AD groups. The AD group included patients carrying *COL1A1, COL1A2, IFITM5,* and *P4HB* mutations, and patients with other gene mutations were classified into the non-AD group. Based on different effects of pathogenic mutations on type I collagen metabolism, the mutations causing amino acid substitutions in the triple helix domain of *COL1A1* or *COL1A2* were classified as collagen structural defects, and nonsense mutations or frame-shift mutations in *COL1A1* or *COL1A2* that led to an early stop codon were classified as collagen protein reducers [20, 23]. Other mutations, such as splicing mutations, were not included because of the difficulty in predicting their effects on type I collagen metabolism.

### Statistical analysis

The Shapiro‒Wilk test and Kolmogorov‒Smirnov test were used to determine whether the data fit a normal distribution. Normally distributed data were expressed as the mean ± standard deviation, abnormally distributed data were expressed as the median (quartiles), and count data were expressed as numbers. Normally distributed data were compared between two groups and among different subgroups with independent sample t-tests and analysis of variance (ANOVA), respectively. Abnormally distributed data were compared between two groups and among more than two groups using the Mann‒Whitney U test and a nonparametric test (Kruskal‒Wallis test), respectively. The chi-square test and Fisher’s exact test were used to compare categorical variables. To explore the correlation, Pearson correlation analysis was applied for normally distributed data, while Spearman correlation analysis was used for abnormally distributed data.

A two-tailed *P* value less than 0.05 was considered statistically significant. Statistical analysis was performed using SPSS software version 25.0 (SPSS, Inc., Chicago, IL, USA). Graphics were drawn using GraphPad Prism software 10.0 (GraphPad, San Diego, CA, USA).

## Results

### Basic characteristics of OI children

A total of 62 children with OI, with an average age of 9.50 (4.86, 12.00) years, were enrolled in this study. 29 age matched healthy children [8.00 (5.50, 10.00) years] were included in the study as normal controls (Table 1). There were 46 boys and 16 girls in OI group, and 13 boys and 16 girls in control group. There was a difference in gender ratio between the two groups (*P* < 0.01) (Table 1). The height of OI children was 128.78 ± 23.92 cm, similar to that of healthy children (129.98 ± 18.61 cm). The height Z score for OI children was -0.49 ± 1.38, which was lower than that for the healthy controls (0.48 ± 1.23, *P* < 0.01) (Table 1).

**Table 1:** Basic characteristics and serum DKK1 level of OI children and healthy controls

	OI (n = 62)	Healthy control (n = 34)	*P* value	Reference range
Gender (male/female)	46/16	13/16	**0.006**	−
Age, years	9.50 (4.86, 12.00)	8.00 (5.50, 10.00)	0.397	−
Height, cm	128.78 ± 23.92	129.98 ± 18.61	0.832	−
Height Z score	− 0.49 ± 1.38	0.48 ± 1.23	**0.005**	−
Weight, kg	30.50 (16.75, 45.25)	25.00 (19.50, 31.00)	0.368	−
Weight Z score	− 0.06 (− 0.74, 1.01)	− 0.14 (− 0.80, 0.79)	0.700	−
Ca, mmol/L	2.48 ± 0.08	2.45 ± 0.08	0.206	2.13–2.70
P, mmol/L	1.69 ± 0.20	1.66 ± 0.16	0.550	0.95–2.65 [49]
ALP, U/L	319.15 ± 95.78	229.58 ± 66.30	**0.000**	42–390 [49]
ALT, U/L	13.50 (10.00, 20.00)	15.00 (10.00, 17.00)	0.823	7–40
Cr, μmol/L	36.50 (30.75, 40.00)	45.00 (33.00, 46.00)	**0.003**	45–84
PTH, pg/mL	24.15 (18.75, 35.60)	17.30 (15.00, 22.30)	**0.000**	15.0–65.0
P1NP, ng/mL	380.50 (273.75, 571.50)	423.00 (343.00, 531.00)	0.507	30.0–3000.0 [50]
β-CTX, ng/mL	1.14 (0.83, 1.43)	1.16 (0.97, 1.41)	0.655	0.40–3.30 [50]
25OHD, ng/mL	22.00 (16.45, 33.15)	23.50 (19.50, 28.65)	0.789	> 30
OPG, ng/mL	0.64 (0.44, 1.05)	0.74 (0.52, 1.35)	0.254	−
Sclerostin, pmol/L	20.87 (14.94, 27.36)	30.41 (22.51,34.00)	**0.001**	−
DKK1, ng/mL	5.20 (4.54, 6.32)	4.08 (3.59, 4.92)	**0.000**	−

The results for normally distributed data were presented as the mean ± SD

Nonnormally distributed data were presented as medians (quartiles)

Bold numbers represent* P* < 0.05

*OI* osteogenesis imperfecta, *Ca* calcium, *P* phosphorus, *ALP* alkaline phosphatase, *ALT* glutamic-pyruvic transaminase, *Cr* creatinine, *PTH* parathyroid hormone, *P1NP* procollagen type 1 N-peptide, *β-CTX* β-C-terminal telopeptide of type 1 collagen, *25OHD* 25-hydroxyvitamin D, *OPG* Osteoprotegerin

According to the Sillence classification, the children with OI were divided into the following groups: type I (34 patients, 55%), type III (12 patients, 19%), and type IV (16 patients, 26%) (Table 2). The annual incidence of peripheral fractures of children with types III and IV OI was higher than those of type I OI, with specific rates being 1.00 (1.00, 2.88) and 1.16 (0.85, 2.30) fractures per year in types III and IV OI versus 0.64(0.30, 1.00) fractures per year in type I OI (all *P* < 0.05) (Table 2). Additionally, the proportion of children with long bone malformations of types III and IV OI was significantly higher than that of type I OI children, accounting for 75% and 31% in types III and IV OI versus 3% in type I OI (all *P* < 0.05) (Table 2). Furthermore, the prevalence of scoliosis of type IV OI children (19%) was obviously higher than type I OI children (0%) (*P* < 0.05) (Table 2).

**Table 2:** The characteristics and serum DKK1 level of different clinical types of OI patients and healthy controls

	OI type I (n = 34)	OI type III (n = 12)	OI type IV (n = 16)	Control (n = 29)	*P* value
Gender (male/female)	22/12	11/1^j^	13/3	13/16	**0.013**
Age, years	10.00 (7.00, 12.00)	4.75 (3.00, 12.50)	8.50 (4.25, 11.00)	8.00 (5.50, 10.00)	0.272
Height, cm	135.63 ± 20.90	117.96 ± 26.34	122.34 ± 24.73	129.98 ± 18.61	0.061
Height Z score	− 0.12 ± 1.00	− 0.91 ± 1.62^j^	− 0.95 ± 1.69^f^	0.48 ± 1.23	**0.003**
Weight, kg	35.00 (22.25, 46.25)	17.00 (13.63, 48.75)	26.00 (15.25, 40.63)	25.00 (19.50, 31.00)	0.205
Weight Z score	0.16 (-0.59, 1.04)	− 0.33 (− 0.75, 0.81)	0.06 (− 1.16, 1.18)	− 0.14 (− 0.80, 0.79)	0.756
Ca, mmol/L	2.48 ± 0.07	2.45 ± 0.06	2.50 ± 0.10	2.45 ± 0.08	0.221
P, mmol/L	1.72 ± 0.18	1.65 ± 0.23	1.65 ± 0.21	1.66 ± 0.16	0.586
ALP, U/L	319.32 ± 87.25^e^	295.42 ± 69.88	336.56 ± 127.30^f^	229.58 ± 66.30	**0.000**
ALT, U/L	13.50 (10.75, 195.00)	11.50 (10.00, 21.50)	19.00 (11.25, 20.00)	15.00 (10.00, 17.00)	0.445
Cr, μmol/L	37.50 (33.00, 43.00)	33.00 (27.50, 37.75)^j^	33.50 (27.50, 39.75)^k^	45.00 (33.00, 46.00)	**0.007**
PTH, pg/mL	27.95 (20.25, 37.10)^b^	20.20 (17.83, 34.18)	20.85 (17.75, 25.85)	17.30 (15.00, 22.30)	**0.000**
P1NP, ng/mL	403.00 (299.00, 577.00)	410.00 (295.00, 758.00)	280.00 (228.70, 506.93)	423.00 (343.00, 531.00)	0.248
β-CTX, ng/mL	1.23 (1.05, 1.62)^a^	1.11 (0.90, 1.30)	0.80 (0.66, 0.87)^k^	1.16 (0.97, 1.41)	**0.000**
25OHD, ng/mL	21.90 (17.25, 28.00)	26.20 (16.25, 58.70)	21.95 (15.70, 42.05)	23.50 (19.50, 28.65)	0.700
LS BMD, g/cm^2^	0.55 (0.48, 0.73)^g, h^	0.41 (0.33, 0.50)	0.37 (0.33, 0.56)	–	**0.001**
LS BMD Z-score	− 0.93 ± 1.26^ g^^, h^	− 2.31 ± 1.54	− 2.31 ± 1.95	–	**0.003**
FN BMD, g/cm2	0.54 ± 0.13^ g^^,h^	0.42 ± 0.12	0.43 ± 0.18	–	**0.013**
FN BMD Z-score	− 2.16 (− 3.45, -1.51)^g, h^	− 4.01 (− 5.02, − 2.06)	− 3.45 (− 5.25, − 2.25)	–	**0.027**
Troch BMD, g/cm^2^	0.41 ± 0.10	0.35 ± 0.12	0.34 ± 0.16	–	0.104
TH BMD, g/cm^2^	0.54 ± 0.12^g^^,h^	0.45 ± 0.12	0.46 ± 0.18	–	**0.048**
OPG, ng/mL	0.71 (0.46, 1.14)	0.61 (0.48, 1.09)	0.55 (0.29, 0.99)	0.74 (0.52, 1.35)	0.314
Sclerostin, pmol/L	25.12 (19.65, 32.46)^c^	17.74 (14.00, 22.61)^l^	18.49 (14.46, 20.52)^m^	30.41 (22.51, 34.00)	**0.000**
DKK1, ng/mL	5.20 (4.61, 6.19) ^e^	5.67 (4.29, 6.42)^j^	4.80 (4.06, 6.48)	4.08 (3.59, 4.92)	**0.003**
Blue sclera, n	28	12	13	–	0.340
Dentinogenesis imperfecta, n	3	2	2	–	0.656
Ligament laxity, n	14	5	6	–	0.965
Hearing abnormality, n	0	0	1	–	0.452
Age at first fracture, years	3.00 (1.00, 5.00)	1.75 (1.00, 4.50)	2.00 (1.05, 5.00)	–	0.534
Peripheral fracture, n	30	12	16	–	0.298
Number of peripheral fracture	3.00 (1.00, 4.50)	4.50 (3.00, 6.00)	3 (2.25, 5.00)	–	0.090
Frequency of peripheral fracture per year	0.64 (0.30, 1.00)^c, d^	1.00 (1.00, 2.88)	1.16 (0.85, 2.30)	–	**0.000**
VCF, n	11	3	8	–	0.334
Wheelchair dependence, n	7	4	4	–	0.619
Long bone deformity, n	1^g^^, h^	9	5	–	**0.000**
Scoliosis, n	0^h^	0	3	–	**0.021**
Ribcage deformity, n	2	2	2	–	0.449

The results for normally distributed data were presented as the mean ± SD

Nonnormally distributed data were presented as medians (quartiles)

Categorical data were presented as numbers

Bold values indicated significant differences among 3 or 4 groups

*OI* osteogenesis imperfecta, *Ca* calcium, *P* phosphorus, *ALP* alkaline phosphatase, *ALT* glutamic-pyruvic transaminase, *Cr* creatinine, *PTH* parathyroid hormone, *P1NP* procollagen type 1 N-peptide, *β-CTX* β-C-terminal telopeptide of type 1 collagen, *25OHD* 25-hydroxyvitamin D, *LS* lumbar spine, *FN* femoral neck, *TH* total hip, *BMD* bone mineral density, *OPG* Osteoprotegerin, *VCF* vertebral compression fracture

^a^*P* < 0.001 for OI-I vs. OI-IV

^b^*P* < 0.001 for OI-I vs. control

^c^*P* < 0.01 for OI-I vs. OI-III

^d^*P* < 0.01 for OI-I vs. OI-IV

^e^*P* < 0.01 for OI-I vs. control

^f^*P* < 0.01 for OI-IV vs. control

^g^*P* < 0.05 for OI-I vs. OI-III

^h^*P* < 0.05 for OI-I vs. OI-IV

^i^*P* < 0.05 for OI-I vs. control

^j^*P* < 0.05 for OI-III vs. control

^k^*P* < 0.05 for OI-IV vs. Control

^l^*P* < 0.01 for OI-II vs. Control

^m^*P* < 0.001 for OI-III vs. OI-IV

**Fig. 1:**
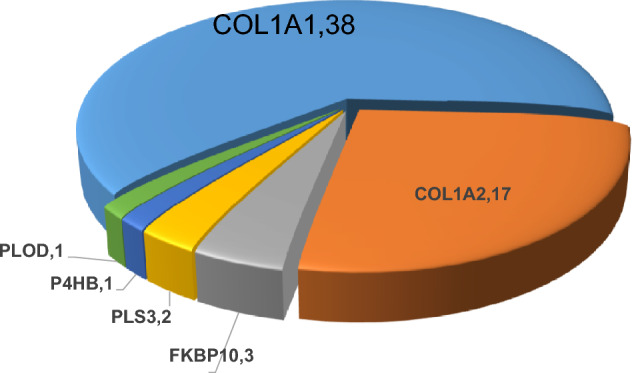
Gene mutation spectrum of OI patients. *OI* osteogenesis imperfecta

The pathogenic mutation spectrum of OI patients in this study was as follows: *COL1A1* (38/62, 61%), *COL1A2* (17/62, 27%), *FKBP10* (3/62, 5%), *PLS3* (2/62, 3%), *P4HB* (1/62, 2%), and *PLOD2* (1/62, 2%) (Fig. 1).

### Serum DKK1 levels and bone turnover marker levels in children with OI

**Fig. 2:**
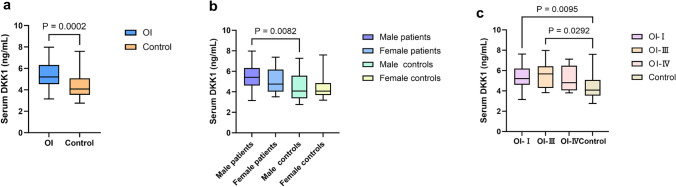
Serum DKK1 concentration in OI children and healthy controls. **a** Serum DKK1 concentration in OI children and healthy children. **b** Serum DKK1 level in OI children and healthy children with different genders. **c** Serum DKK1 level in different clinical types of OI children and healthy controls. Significant differences between two groups were indicated in brackets. *OI* osteogenesis imperfecta

The serum DKK1 concentration was 5.20 (4.54, 6.32) ng/mL in children with OI, which was significantly higher than that of healthy children [4.08 (3.59, 4.92)] ng/mL (*P* < 0.001) (Fig. 2a). No difference was found in the serum DKK1 concentrations between boys and girls with OI (Fig. 2b). The serum DKK1 concentration was 5.20 (4.61, 6.19) ng/mL, 5.67 (4.29, 6.42) ng/mL and 4.80 (4.06, 6.48) ng/mL in children with type I, type III OI and type IV OI, respectively (Table 2). No significant difference in the serum DKK1 levels was observed among different clinical types of OI children (Fig. 2c). The serum DKK1 concentration in type I and type III OI children was significantly higher than that in normal children (all *P* < 0.05) (Fig. 2c).

The serum ALP level in children with OI was 319.15 ± 95.78 U/L, which was significantly higher than that in healthy children (229.58 ± 66.30 U/L) (*P* < 0.001). There were no significant differences in the serum 25-hydroxyvitamin D, calcium, phosphorus, β-CTX and P1NP levels between children with OI and healthy controls (Table 1). The serum sclerostin level in children with OI was 20.87 (14.94, 27.36) pmol/L, which was significantly lower than that in healthy children [30.41 (22.51, 34.00) pmol/L] (*P* < 0.01). There were no significant differences in the OPG level between children with OI and healthy controls (Table 1).

### Correlation between serum DKK1 level and skeletal phenotype in OI children

**Fig. 3:**
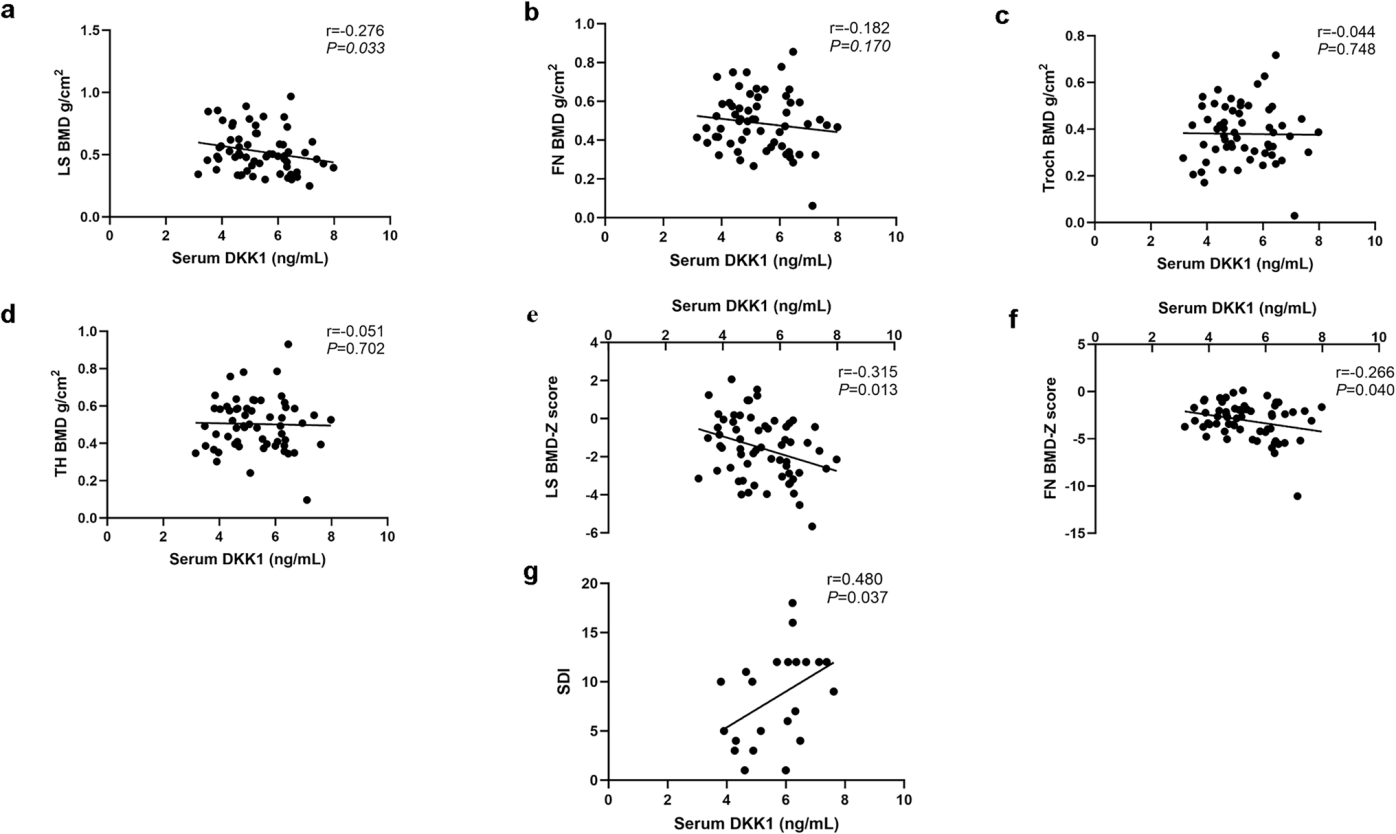
Relationships between serum DKK1 level and BMD, BMD Z score and Spinal Deformity Index in OI children. **a** Correlation between serum DKK1 level and LS BMD in OI children. **b** Correlation between serum DKK1 level and FN BMD in OI children. **c** Correlation between serum DKK1 level and trochanter BMD in OI children. **d** Correlation between serum DKK1 level and TH BMD in OI children. **e** Correlation between serum DKK1 level and LS BMD Z score in OI children. **f** Correlation between serum DKK1 level and FN BMD Z score in OI children. **g** Correlation between serum DKK1 level and Spinal Deformity Index (SDI) in OI children with spinal deformities. *OI* osteogenesis imperfecta, *LS* lumbar spine, *FN* femoral neck, *TH* total hip, *BMD* bone mineral density, *SDI* spinal deformity index

After adjusting for age and gender, the serum DKK1 concentration in OI children exhibited a significantly negative correlation with lumbar spine BMD (r = − 0.276,* P* < 0.05) (Fig. 3a). However, no significant correlations were observed between DKK1 concentration and BMD at the femoral neck, greater trochanter, or total hip (Fig. 3b–d). Furthermore, there was a negative correlation between serum DKK1 concentration and BMD Z scores at LS and FN (r = − 0.315, *P* < 0.05; r = − 0.266, *P* < 0.05) (Fig. 3e, f).

**Fig. 4:**
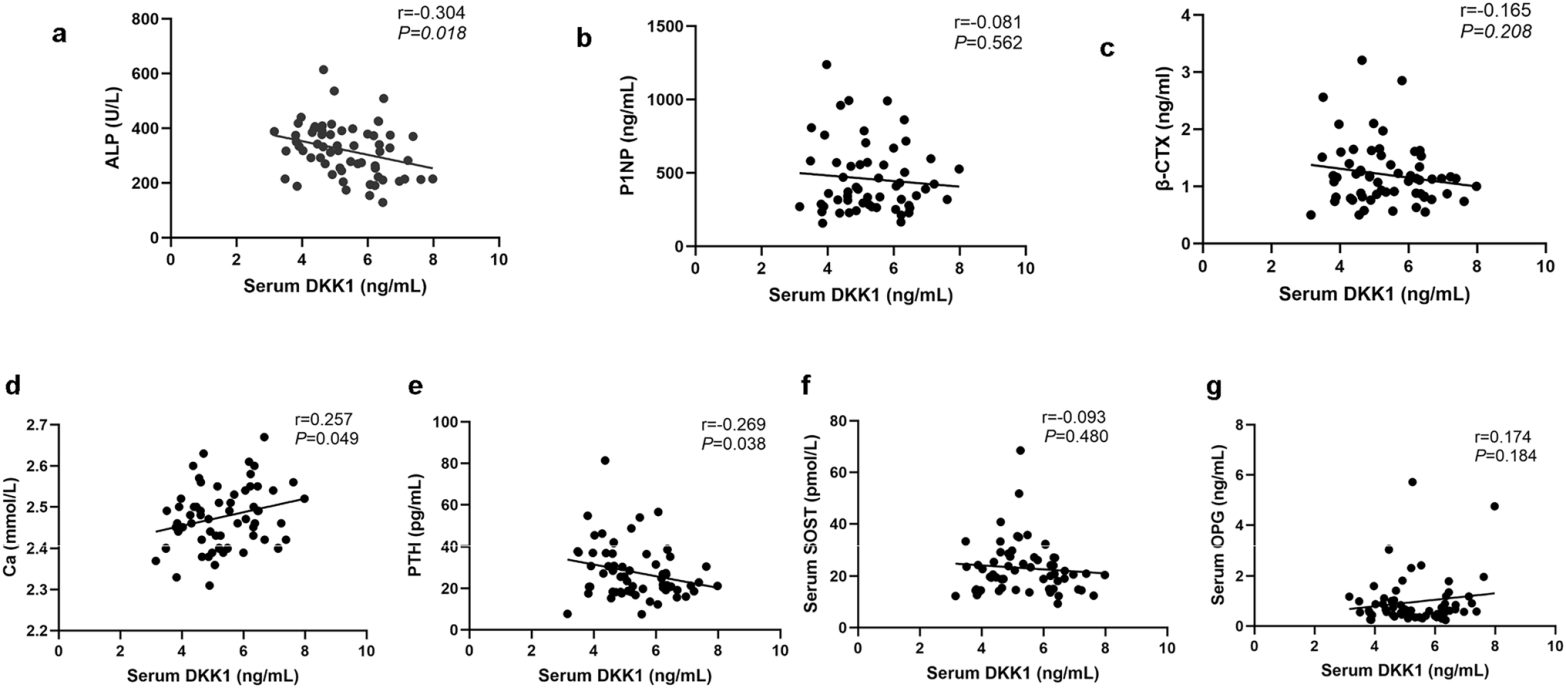
Relationships between serum DKK1 level and bone turnover markers in OI children. **a** Correlation of serum DKK1 level with ALP level in OI children. **b** Correlation of serum DKK1 level with P1NP level in OI children. **c** Correlation of serum DKK1 level with β-CTX level in OI children. **d** Correlation of serum DKK1 level with Ca level in OI children. **e** Correlation of serum DKK1 level with PTH level in OI children. **f** Correlation of serum DKK1 level with SOST level in OI children. **g** Correlation of serum DKK1 level with OPG level in OI children. *OI* osteogenesis imperfecta, *ALP* alkaline phosphatase, *P1NP* procollagen type 1 N-peptide, *β-CTX* β-C-terminal telopeptide of type 1 collagen, *Ca* calcium, *PTH* parathyroid hormone, *SOST* sclerostin, *OPG* osteoprotegerin

**Fig. 5:**
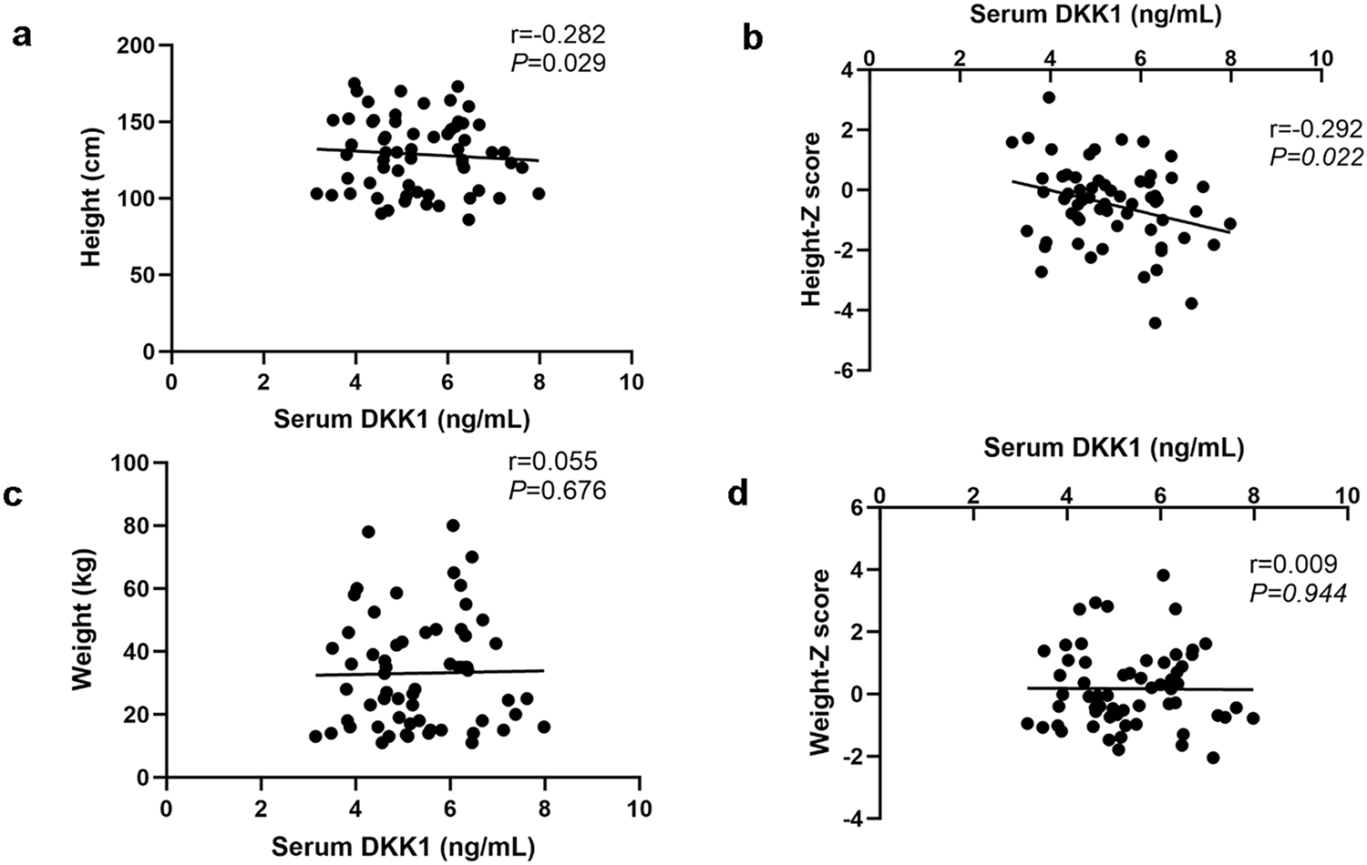
Relationships between serum DKK1 level and height, weight and their Z scores in OI children. **a** Correlation between serum DKK1 level and height in OI children. **b** Correlation between serum DKK1 level and height Z score in OI children. **c** Correlation between serum DKK1 level and weight in OI children. **d** Correlation between serum DKK1 level and weight Z score in OI children. *OI* osteogenesis imperfecta

The serum DKK1 concentration in OI children was negatively correlated with the ALP concentration (r = − 0.304, *P* < 0.05) (Fig. 4a) and positively correlated with serum calcium concentration (r = 0.257,* P* < 0.05) (Fig. 4d) and negatively correlated with PTH concentration (r = − 0.269, *P* < 0.05) (Fig. 4e). The serum DKK1 concentration in children with OI was negatively correlated with height (r = − 0.282,* P* < 0.05) and the height Z score (r = − 0.292, *P* < 0.05) (Fig. 5a, b). No significant correlations were found between serum DKK1 level and age, weight, weight Z score, serum levels of P1NP, β-CTX, P, 25-hydroxyvitamin D, sclerostin and OPG or liver and kidney function of children with OI (Fig. 4b, c, f, g, Fig. 5c, d and Supplementary Fig. 1a–b).

There was no significant correlation between the serum DKK1 concentration and annual peripheral fractures incidence of children with OI (Supplementary Fig. 1c). The serum DKK1 concentrations were 5.67 ± 1.12 ng/mL and 5.19 ± 1.12 ng/mL in OI patients with and without vertebral compression fractures, respectively, which had no significant difference (Table 3). In patients with spinal deformity (22/62), serum DKK1 concentration was positively correlated with SDI (r = 0.480, *P* < 0.05) (Fig. 3g).

**Table 3:** Serum DKK1 level in OI patients with different genetic mode, abnormal collagen metabolism, and spinal fracture or not

	DKK1, ng/mL	*P* value
Different genders		
Boys	5.44 ± 1.10	0.342
Girls	5.12 ± 1.26	
Different genetic modes		
AD (n = 56)	5.35 ± 1.18	0.799
Non-AD (n = 6)	5.47 ± 0.65	
Abnormal collagen metabolism		
Structural defect (n = 25)	5.57 ± 1.27	0.359
Quantitative reduction (n = 25)	5.26 ± 1.14	
Vertebral compression fractures		
VCF (n = 22)	5.67 ± 1.12	0.113
Non-VCF (n = 40)	5.19 ± 1.12	

The results for normally distributed data were presented as the mean ± SD

Nonnormally distributed data were presented as medians (quartiles)

*AD* autosomal dominant inheritance, *VCF* vertebral compression fracture

### Relationship between serum DKK1 level and genotype in OI children

According to the pathogenic gene mutation profile, 56 patients consisted with AD inheritance, 4 with AR inheritance, and 2 with X-linked inheritance. The serum DKK1 concentrations of OI children had no significant difference between the AD group and the non-AD group. Based on abnormal metabolic patterns of type I collagen, there were 25 patients with collagen structural defects (8 patients with α1 chain defects and 17 patients with α2 chain defects) and 25 patients with reduced collagen quantity. No significant difference was found between OI children with structural defects and insufficient quantity of type I collagen (Table 3).

## Discussion

This study detected serum DKK1 level in a relatively large cohort of children with OI and assessed the correlation for the first time between DKK1 level and the skeletal phenotype and genotype in OI children. We found that serum DKK1 level of OI children was significantly higher than that of age-matched normal children. Interestingly, the serum DKK1 concentration was negatively correlated with the bone formation biomarker ALP, lumbar spine BMD and BMD Z scores at lumbar spine and femoral neck, and serum DKK1 concentration was positively correlated with SDI in OI children with spinal deformities. No significant correlation was found between DKK1 level and fracture incidence or OI pathogenic genotype. These findings indicated DKK1 may be a useful novel biomarker for OI.

A series of studies have shown that DKK1 plays important roles in regulating bone formation as it is the natural inhibitor of the WNT signaling pathway. DKK1 had been found to be associated with many skeletal diseases [24–29]. In a 3-year follow-up study of liver transplant recipients, serum DKK1 level was significantly increased, and liver transplant recipients who experienced fractures had significantly higher DKK1 level than patients without fractures [25]. In patients with multiple myeloma, the level of DKK1 was significantly increased, and the severity of skeletal lesions was significantly positively correlated with the DKK1 level [26]. The mechanism involved that DKK1 could be secreted by multiple myeloma cells and bone marrow mesenchymal stem cells, which inhibited the classical WNT pathway, impeding osteoblast maturation and bone matrix mineralization, leading to osteolytic lesions of multiple myeloma [27]. In addition, a murine model of breast cancer had unveiled that DKK1 overexpression markedly enhanced bone metastasis and osteolysis, concurrently upregulated tumor proliferation within metastatic sites, and the knockdown of DKK1 could mitigate bone metastasis [28]. Moreover, patients with disuse osteoporosis due to long-term bed rest had elevated serum DKK1 level and reduced expression of β-catenin, resulting in decreased bone formation and increased bone resorption [29]. The above studies indicated that DKK1 is an important factor regulating bone turnover, and its abnormal secretion is closely related to various metabolic and tumorigenic bone diseases.

There were also studies on DKK1 in small sample of patients with OI and animal model of OI. In a study involving 18 OI children, the serum DKK1 level was higher in OI group than in the age-matched normal control group [30]. Furthermore, the study revealed that the serum from OI children could inhibit differentiation of osteoblasts, and this effect could be countered by an anti-DKK1 antibody [30]. In addition, animal studies revealed that the expression of DKK1 was higher and β-catenin was lower in bone tissues of OI model (OIM) mice [31]. DKK1 expression could be significantly inhibited after treatment with antisense oligonucleotides that antagonize microRNA-29a, and bone microarchitecture and BMD of OIM mice were improved [31]. Based on the findings of the above study and our study, DKK1 may regulate bone formation through affecting the WNT pathway, thereby participating in the pathological processes of OI.

As we know, the WNT pathway plays an essential role in osteoblast differentiation and maturation [32, 33], which is initiated when the WNT ligand binds to the Frizzled and LRP5/6 receptors simultaneously. The activation of co-receptors leads to the inhibition of glycogen synthase kinase 3 (Gsk3) activity and the stabilization of the β-catenin protein [34]. Stable β-catenin subsequently undergoes nuclear translocation and interacts with T-cell factor and lymphoid enhancer factor (TCF/LEF), a transcription factor, to promote gene expression in osteoblasts [34, 35]. DKK1 is a natural antagonist of the WNT pathway. Our studies demonstrated that serum DKK1 concentration was significantly higher in OI patients, which could inhibit the activity of the WNT pathway, leading to a decrease in the expression of multiple genes in osteoblasts, reduce differentiation and maturation of osteoblasts, and inhibit bone formation. Therefore, we found that DKK1 level was negatively correlated with bone formation biomarker of ALP and lumbar BMD, Z scores of BMD at lumbar spine and femoral neck, indicating that DKK1 had the potential as a novel biochemical marker for OI patients.

Moreover, the natural WNT antagonist, sclerostin, has become a important target of anti-osteoporosis drugs. Romosozumab, a monoclonal antibody of sclerostin, is effective in increasing BMD and reducing vertebral, nonvertebral, and hip fractures [36, 37]. It has shown potential therapeutic benefits in two patients with OI [38, 39]. Moreover, setrusumab, another monoclonal antibody of sclerostin, has exhibited promising treatment potential in a randomized Phase IIb study in adults with OI and in a phase 2/3 ORBIT study involving children and adolescents with OI [40, 41]. Furthermore, sclerostin nucleic acid aptamers targeting the Loop3 domain of sclerostin, can promote bone formation, increase BMD, and improve bone microarchitecture of OI animal model [42]. Notably, sclerostin antibodies have shown promising outcomes in various mouse models mimicking OI [43–45]. These findings indicate that natural antagonists of the WNT pathway may hold immense potential in the treatment of OI.

It is worth noting that DKK1 is also natural antagonist of WNT pathway, which is expected to be a novel therapy target for OI. Studies showed that a reduction in DKK1 level through heterozygous gene knockout could lead to an increase in bone formation, vertebral trabecular bone volume and trabecular thickness in ovariectomized (OVX) mice [46]. The deletion of DKK1 could increase bone formation through resulting in an elevated skeletal expression of WNT target genes, including *Lef1* and *Axin2* [46]. In a murine model of multiple myelom, anti-DKK1 antibody could prevent bone loss in both trabecular and cortical compartments [47]. Moreover, animal experiments demonstrated that DKK1-Ab at a dose of 25 mg/kg twice a week for 28 days could enhance callus formation and bone mechanical strength in a tibial fracture mouse model [48]. These studies suggested that DKK1 may also be one of the potential targets for treatment of OI.

This study indicated that serum DKK1 level was higher in OI children than that in healthy children. Serum DKK1 level was found for the first time to be negatively correlated with serum ALP level, lumbar BMD, BMD Z scores at lumbar spine and femoral neck in children with OI. DKK1 level was also positively correlated with SDI in OI children with spinal deformities. However, this study had a series of limitations: only serum DKK1 concentration was measured, and we did not measure the DKK1 level in bone tissue, which is more important for regulating bone formation. In addition, this study was a cross-sectional design, which only indicated some correlations, but could not confirm their causal relationships. The sample size of this study was relatively small, and it was difficult to reveal the correlation between DKK1 level and fracture incidence or genotype of OI patients.

## Conclusion

The serum DKK1 level was not only significantly elevated in OI children, but also closely correlated to their skeletal phenotype, suggesting that DKK1 may become a novel biomarker and a potential therapeutic target of OI.

## Supplementary Information

Below is the link to the electronic supplementary material.

Supplementary file1 (PPTX 658 kb)

## Data Availability

The datasets generated and/or analysed during the current study are not publicly available due to the protection of patient confidentiality but are available from the corresponding author on reasonable request.
