# Supplementary material for: Correlation of serum DKK1 level with skeletal phenotype in children with osteogenesis imperfecta
Source: J Endocrinol Invest. 2024 May 14;47(11):2785–95. doi: 10.1007/s40618-024-02380-9 (PMC11473575; doi:10.1007/s40618-024-02380-9)
Supplement: Supplementary file 1 — Supplementary file1 (PPTX 658 kb) [file 40618_2024_2380_MOESM1_ESM.pptx]

## Slide 1
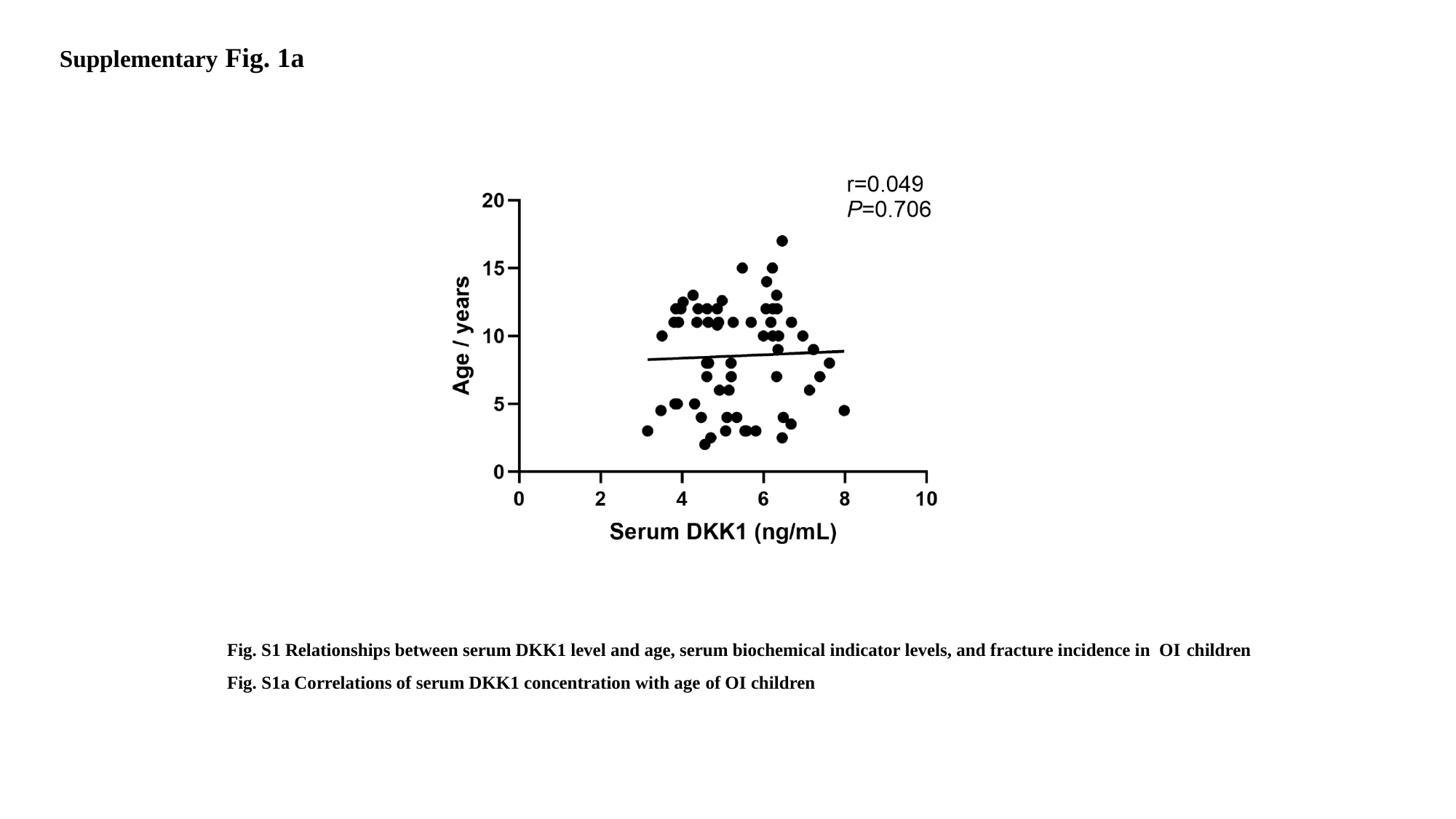

Supplementary Fig. 1a
Fig. S1 Relationships between serum DKK1 level and age, serum biochemical indicator levels, and fracture incidence in OI children
Fig. S1a Correlations of serum DKK1 concentration with age of OI children

## Slide 2
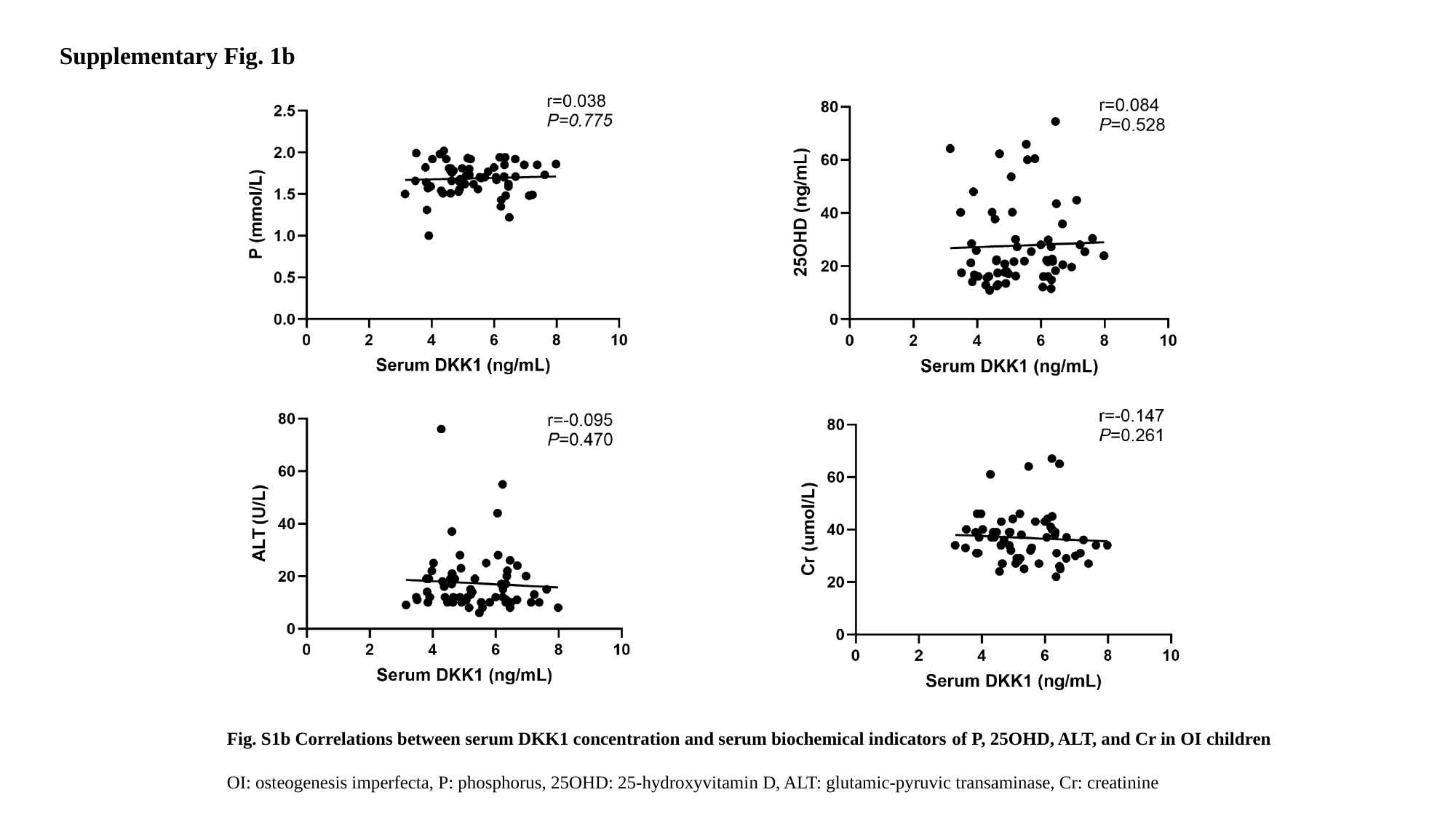

Supplementary Fig. 1b
Fig. S1b Correlations between serum DKK1 concentration and serum biochemical indicators of P, 25OHD, ALT, and Cr in OI children
OI: osteogenesis imperfecta, P: phosphorus, 25OHD: 25-hydroxyvitamin D, ALT: glutamic-pyruvic transaminase, Cr: creatinine

## Slide 3
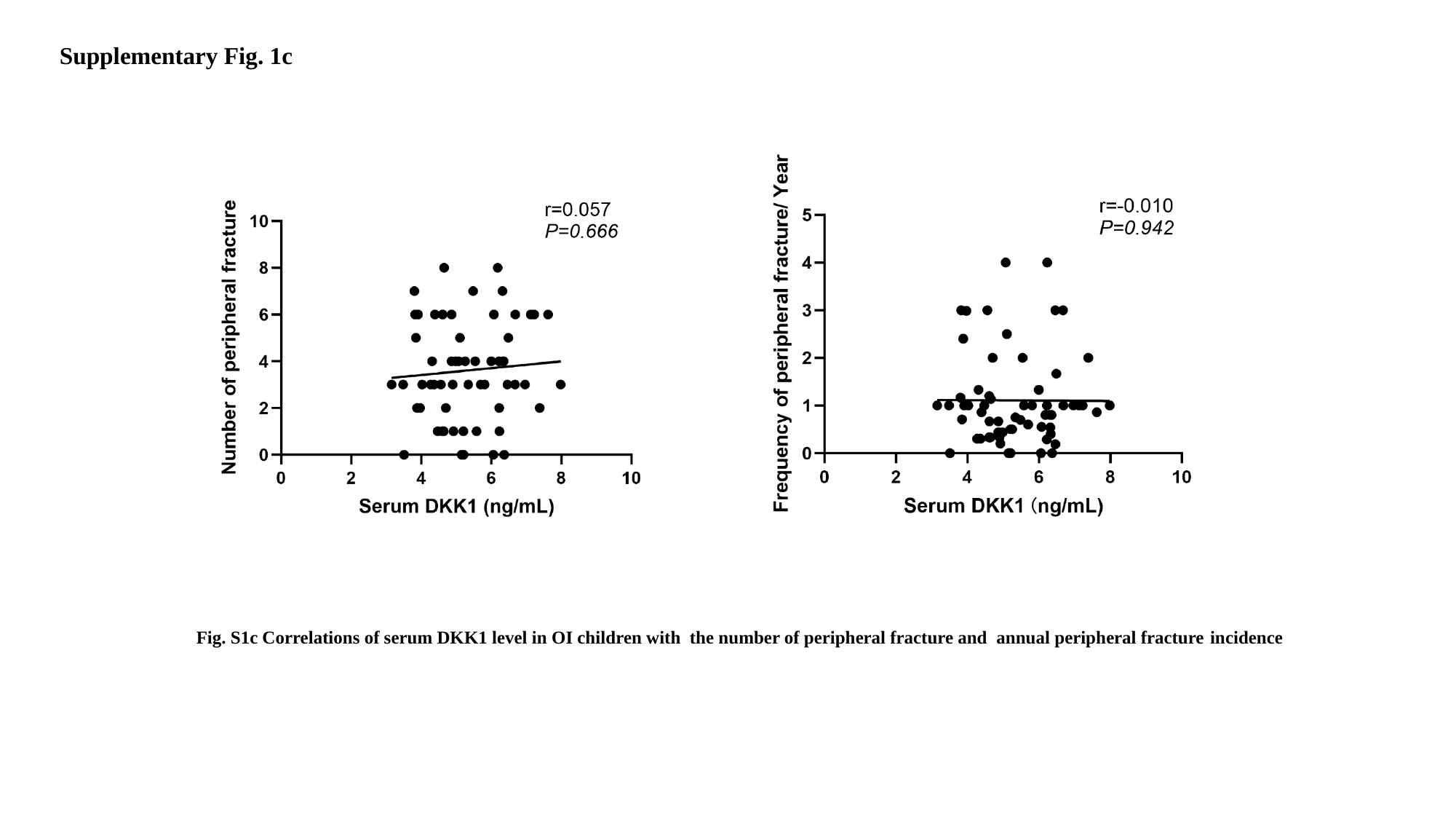

Supplementary Fig. 1c
Fig. S1c Correlations of serum DKK1 level in OI children with the number of peripheral fracture and annual peripheral fracture incidence
